# Type 2 Diabetes Mellitus Diagnosed at the Age of Eight Years: A Case Report

**DOI:** 10.7759/cureus.85065

**Published:** 2025-05-29

**Authors:** Md Rakibul Hasan, Mashfiqul Hasan, Al Aharama

**Affiliations:** 1 Endocrinology and Diabetes, Medical College for Women and Hospital, Dhaka, BGD; 2 Endocrinology and Diabetes, Bangabandhu Sheikh Mujib Medical University, Dhaka, BGD; 3 Biochemistry, Medical College for Women and Hospital, Dhaka, BGD

**Keywords:** childhood diabetes, diabetes, prepubertal type 2 diabetes mellitus, type 2 diabetes in children and adolescents, young onset diabetes

## Abstract

The onset of type 2 diabetes mellitus (T2DM) in prepubertal age is not uncommon nowadays. Here we are reporting on a young boy who was diagnosed with diabetes at the age of eight years. Diagnosis of diabetes was made based on symptoms of hyperglycemia (new onset bed wetting, also polyphagia and polydipsia) and laboratory evidence of high plasma glucose. The patient was obese (body mass index, BMI > 95th percentile) and had acanthosis nigricans during presentation. The type of diabetes was not confirmed at the time of diagnosis based on the supporting laboratory investigations. His initial high blood glucose was managed with a basal bolus insulin regimen with symptomatic improvement. Due to poor follow-up and inadequate adherence to a complex insulin regimen, his blood glucose was not well controlled. Sometimes the patient stopped insulin for several weeks without evidence of hyperglycemic crisis. We excluded type 1 diabetes by checking fasting C-peptide and islet autoantibodies. He had a strong family history of diabetes at a very young age of onset, affecting all generations. Both parents had diabetes, and he was exposed to high maternal blood glucose during gestation. He was delivered by cesarean section at 37 completed weeks of gestation, his birth weight was 3.5 kg, and he did not experience post-delivery hypoglycemia, according to the statement of his guardian. Mother was treated with insulin during gestation, but blood glucose control was not satisfactory. All these conditions are considered risk factors of T2DM. However, maturity-onset diabetes of the young (MODY) could be a possible differential diagnosis, considering a strong family history. So, we checked 14 MODY-related genes (GCK, HNF1A, HNF4A, HNF1B, INS, NEUROD1, PDX1, PAX4, ABCC8, KCNJ11, KLF11, CEL, BLK, and APPL1) and found no pathogenic variant. Finally, we confirmed him as a case of type 2 DM. We changed his treatment from a complex basal bolus insulin regimen to twice-daily premixed insulin with continuation of metformin (500 mg, twice daily), which was already prescribed by another physician. On follow-up, his glycated hemoglobin reduced significantly (from 11.1% to 8.0%). As his obesity was an important issue, we initiated semaglutide at a dose of 0.25 mg later on. Metformin was stopped due to dyspepsia. On the last follow-up, the patient lost significant weight with better control of diabetes (ranging from 5.0 to 5.7 mmol/L in a fasting state and post-meal capillary glucose <8.2 mmol/L using a glucometer at home) without any evidence of hypoglycemia after the initiation of semaglutide, and the dose of insulin was reduced significantly. We planned to stop insulin and maintain an ideal body weight while ensuring good compliance with lifestyle advice. We checked diabetes-related macro- and microvascular complications, which were negative, except in one episode, trace albumin was present in urine, which was negative on subsequent investigations.

## Introduction

Globally, more than 70% of deaths are attributed to non-communicable diseases (NCDs). Diabetes is one of the top five NCDs contributing to death in a significant number of the population globally [1]. The onset of type 2 diabetes mellitus (T2DM) generally starts in adulthood. However, several studies [2,3] have reported a higher prevalence of T2DM in children and adolescents, making it a growing concern in the modern world. Many environmental, genetic, and lifestyle factors predispose children to develop early-onset diabetes mellitus. Ethnic difference (Asian origin) is also considered a risk factor. Children with obesity, exposure to maternal high blood glucose during gestation, or a family history of diabetes are at high risk and need screening for DM as early as the onset of puberty or at 10 years of age. The American Diabetes Association (ADA) recommended screening for T2DM and prediabetes in asymptomatic children and adolescents among children and adolescents with these risk factors [4]. The development of T2DM before the age of 10 years has been reported in different parts of the world. A recent cross-sectional study from Bangladesh with 68 T2DM children and adolescents reported that 10 (14.7%) patients were below the age of 10, starting from nine years [5]. Only a few cases of T2DM before the age of nine years are available [6,7], and no cases have been reported from Bangladesh. Here we are reporting a case of T2DM, which was first diagnosed at the age of eight years. To our knowledge, this is the youngest T2DM case report from Bangladesh so far.

## Case presentation

**Figure 1:**
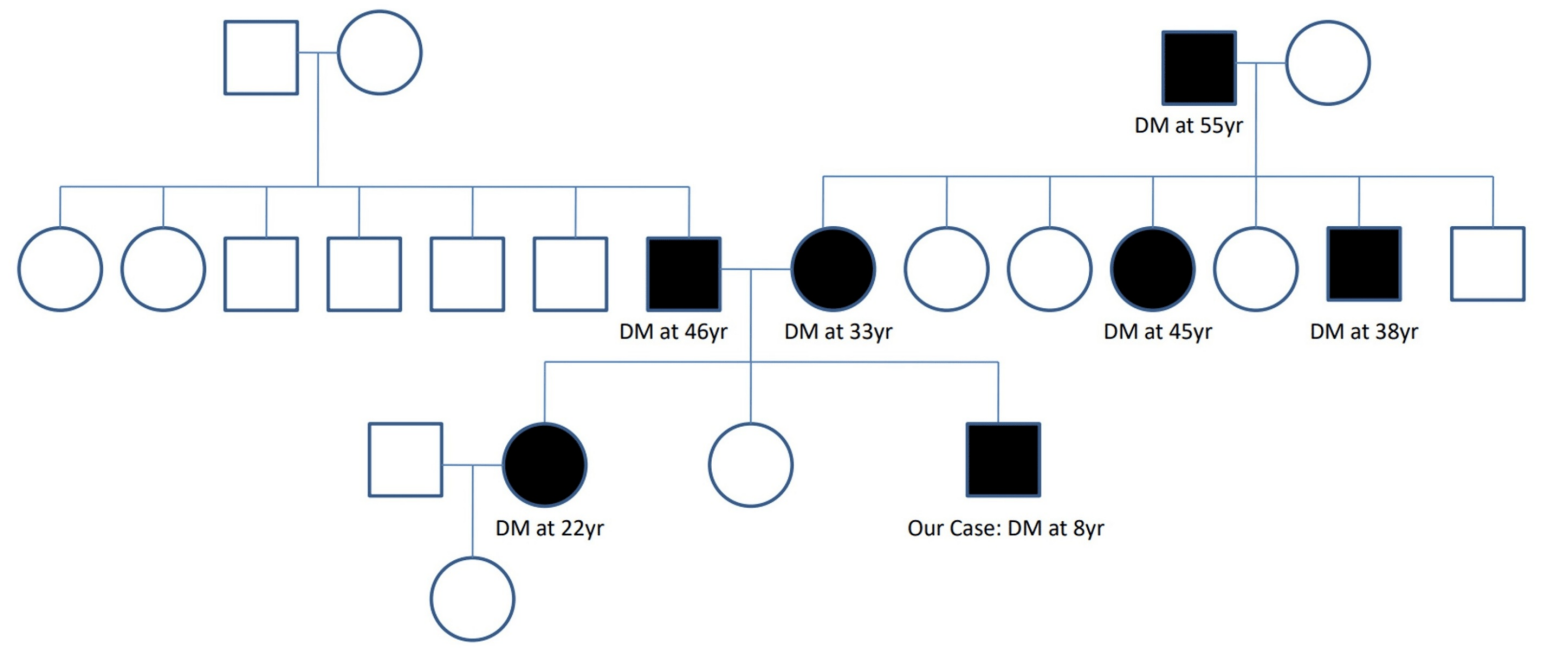
Family pedigree DM: diabetes mellitus Image created by the authors.

We present a 14-year-old boy of Bangladeshi origin who was first diagnosed as a case of DM at the age of eight years in stage one of puberty in 2019. His parents (both mother and father) had established diabetes before his conception. He was exposed to high blood glucose levels during gestation. Out of three siblings, two (including our case) have diabetes. His mother has a strong family history of DM affecting all generations (Figure 1). The patient presented with the reappearance of bedwetting at the age of eight years, which was present up to the age of two years.

He also had polyphagia and polydipsia over the previous three months, but his parents did not pay any attention to it. Parents were more concerned about his new-onset bedwetting and measured his capillary blood glucose at home with a glucometer and found it to be 10.0 mmol/L. He was referred to a physician, where an oral glucose tolerance test (OGTT) confirmed the diagnosis of DM (Table 1). The patient was hospitalized immediately to exclude the possibility of an acute hyperglycemic emergency. Insulin was started as an initial treatment with dramatic improvement of his blood glucose and bedwetting. He was discharged from the hospital within weeks. During hospitalization, his fasting C-peptide was checked and found to be high (Table 1). He was provisionally diagnosed as a case of T2DM, but the possibility of maturity-onset diabetes of the young (MODY) was not excluded due to a strong family history of young-onset DM affecting all generations. At the time of first presentation (in 2019), the patient had a higher body weight of 41 kg (> 97th percentile), a height of 128 cm (50th percentile), and a BMI of 25 kg/m² (> 95th percentile). He had acanthosis nigricans at that time and also has it now. His birth weight was 3.5 kg, delivered at 37 weeks of gestation by cesarean section, with no post-delivery hypoglycemia or any convulsion, according to the patient's guardian. His mother was treated with insulin during gestation; glycemic control was not standard.

**Table 1:** Investigation profile GAD-65: glutamic acid decarboxylase-65; ZnT8: zinc transporter 8; IA-2: islets antigen-2 autoantibody; MODY: maturity-onset diabetes of the young; ESR: erythrocyte sedimentation rate; SGPT: serum glutamate pyruvate transaminase; TSH: thyroid stimulating hormone; LDL: low-density lipoprotein; HDL: high-density lipoprotein

Name of test	Result	Normal value
Investigations at the time of diagnosis (2019)		
Fasting plasma glucose (mmol/L)	10.1	<5.6
2 hours after a 75-gram glucose load (mmol/L)	15.1	<7.8
Hemoglobin (gm/dl)	15.4	12-15.5
ESR (mm in 1^st ^hour)	05	<10
WBC (10^9/L)	10.15	4.5-13.5
Platelet count (10^9/L)	340	150-450
Serum electrolytes (mmol/L)		
Serum sodium	137	135-146
Serum potassium	4.2	3.5-5.3
Serum chloride	101	94-110
Total carbon dioxide (TCO_2_)	26	18-26
Urine routine, microscopy, and examination		
Urine sugar	+++	Nill
Urine protein	Trace	Nill
Urine ketone body	Absent	Nill
SGPT (U/L)	63	<63
Creatinine (mg/dl)	0.79	0.24-.74
C-peptide (ng/ml)	3.67	0.7 to 1.9
Investigation in the fourth year of diagnosis (2023)		
TSH (µIU/ml)	3.23	0.7-5.7
Free T4 (ng/dl)	1.36	0.89-1.72
HbA1C (%)	11.1	<5.7
Fasting lipid profile (mg/dl)		
Total cholesterol	215.78	<170
LDL-cholesterol	139.21	<110
HDL-cholesterol	48.33	>45
Triglycerides	139.99	< 75
USG whole abdomen	Normal study	
Genetic test in the fifth year of diagnosis (2024)		
GAD-65 antibody	Negative	
ZnT8 and IA-2 autoantibody	Negative	
14 MODY related gene analysis (GCK, HNF1A, HNF4A, HNF1B, INS, NEUROD1, PDX1, PAX4, ABCC8, KCNJ11, KLF11, CEL, BLK, APPL1)	No pathogenic variant	

The patient was presented to our endocrine OPD due to poor glycemic control. From the diagnosis of DM, the patient had very fluctuating blood glucose levels with poor compliance to medicine and lifestyle, but had not experienced any episodes of hyperglycemic or hypoglycemic emergency leading to hospitalization. The patient was prescribed a basal bolus insulin regimen with metformin 1000 mg daily. Due to poor compliance and frequent missing doses of lunchtime insulin, we switched to premixed insulin twice daily with continuation of metformin. Proper counseling was done with the patient face-to-face. The last reported HbA1C was 8.0%, which was better compared to the previous one (11.1%) in June 2023. We introduced semaglutide 0.25 mg once weekly in December 2024, along with existing treatment. But due to dyspepsia, we stopped metformin. The patient did not follow up physically for the last four months due to living in a remote area of the countryside. Over the phone call, we confirmed his recent blood glucose is now well-controlled (fasting ranging from 5.0 to 5.7 mmol/L and post-meal capillary glucose <8.2 mmol/L using a glucometer at home) without any evidence of hypoglycemia; the patient lost significant weight after initiation of semaglutide. We evaluated the patient for diabetes-related macrovascular and microvascular complications, all of which were negative, except for one episode in which trace albumin was detected in the urine; however, this finding was not confirmed on subsequent testing. Besides glycemic control, we also looked for confirmation of the types of DM. We checked glutamic acid decarboxylase-65 (GAD-65), zinc transporter 8 (ZnT8), and islet antigen-2 (IA-2) autoantibody, which were found negative. We also screened for variants of 14 MODY-related genes (GCK, HNF1A, HNF4A, HNF1B, INS, NEUROD1, PDX1, PAX4, ABCC8, KCNJ11, KLF11, CEL, BLK, APPL1), where no pathogenic variant could be identified. The genetic screening was done at DNA Solutions Ltd., Dhaka, by using the next-generation sequencing platform of the National Institute of Biotechnology, Savar, Dhaka, Bangladesh. There were no characteristic extra-pancreatic features of MODY. So, we confirmed the diagnosis of T2DM at this very early age.

## Discussion

The incidence of DM in children and adolescents was observed to be 24.3 per 100,000 person-years in a survey done in the USA [2]. Most of the patients were T1DM, irrespective of their ethnicity. The incidence of T2DM below the age of 10 years was negligible compared to T1DM. It was observed to be lowest in non-Hispanic White (0.6%) and highest in Asian Americans/Pacific Islanders (13.6%) [2]. Childhood obesity, intrauterine exposure to maternal high blood glucose, and sedentary lifestyle were all considered important risk factors of childhood T2DM. A recent meta-analysis of 228,184 participants observed that the prevalence of T2DM and prediabetes in children and adolescents was 1.3% and 17% in obese subjects, which was 13 times and three times higher than in normal-weight subjects (0.1% and 3%), respectively [8]. Offspring exposed to high maternal plasma glucose is 7.76 times more likely to develop diabetes [9]. Our patient was exposed to intrauterine high blood glucose due to maternal T2DM, which made our patient more vulnerable to developing DM. However, a strong family history of diabetes at a younger age of onset raises the possibility of MODY. We excluded MODY by checking 14 MODY-related gene mutations, where no pathogenic variant could be identified. At the time of presentation, our patient was obese (BMI > 95th percentile), and there was evidence of insulin resistance (presence of acanthosis nigricans) and very high C-peptide, which excludes the possibility of T1DM. Furthermore, we checked GAD-65 and islet autoantibodies; they were negative, which made the diagnosis of T1DM unlikely. Clinical, metabolic profile, strong family history of DM, and intrauterine exposure to maternal high plasma glucose were similarly found in two case reports of T2DM diagnosed at five and seven years of age [6,7]. Diabetes is already a threat to the healthcare system of developing countries; the early onset of T2DM in the prepubertal age would be a disaster for the nation, as it would add more and more diabetes-related complications at a very early age.

## Conclusions

The paradigm shift of the onset of T2DM from the elderly to children and adolescents is very alarming, which needs more emphasis on diabetes preventive strategies to save the future generation. Optimization of the treatment regime should be considered for young-onset T2DM, considering poor medication adherence. Early and tight glycemic control should be the priority to prevent long-term DM-related complications. Frequent and repeated counseling sessions should be arranged to ensure treatment adherence and to achieve good glycemic control. Psychological issues should also be focused on during follow-up to ensure treatment adherence.
